# Proton Beam Therapy for Lung Oligometastatic Recurrence in Patients With Esophageal Cancer

**DOI:** 10.7759/cureus.50343

**Published:** 2023-12-11

**Authors:** Hisashi Yamaguchi, Takahiro Kato, Michitaka Honda, Koichi Hamada, Yojiro Ishikawa, Ichiro Seto, Yoshiaki Takagawa, Motohisa Suzuki, Yasuhiro Kikuchi, Masao Murakami

**Affiliations:** 1 Department of Minimally Invasive Surgical and Medical Oncology, Fukushima Medical University, Fukushima, JPN; 2 Department of Radiation Oncology, Southern Tohoku Proton Therapy Center, Koriyama, JPN; 3 Department of Surgery, Southern Tohoku General Hospital, Koriyama, JPN; 4 Department of Gastroenterology, Southern Tohoku General Hospital, Koriyama, JPN; 5 Department of Radiology, Tohoku Medical and Pharmaceutical University, Sendai, JPN

**Keywords:** oligometastasis, overall survival (os), isolated lung metastasis, proton beam therapy, esophageal cancer (ec)

## Abstract

Local treatment of oligometastatic esophagogastric cancer has been reported to improve overall survival (OS) compared to systemic therapy alone. This study evaluated the feasibility and safety of proton beam therapy (PBT) for the treatment of lung oligometastatic recurrence in esophageal cancer patients. This single-center historical cohort study enrolled 11 patients who underwent PBT for lung oligometastasis from esophageal cancer between 2010 and 2019. The selection criteria were that the primary esophageal cancer was controlled and no more than three lung metastases without outside lung tumors were present. OS, progression-free survival (PFS), and local control (LC) rates and adverse events (AEs) were assessed. Factors that may be related to OS were also investigated. The median follow-up period was 27.8 months (8.8-141.3 months). The one-, two-, and three-year OS rates were 81.8%, 72.7%, and 51.9%, respectively (median OS time: 43.7 months); PFS rates were 45.5%, 27.3%, and 27.3%, respectively (median PFS time: 8.8 months); and LC rates were 92.3%, 72.7%, and 72.7%, respectively. The eighth edition of tumor-node-metastasis (TNM) classification for esophageal cancer was the only significant OS-related factor (p = 0.0309). No grade ≥ 3 AEs were observed. Based on the low incidence of AEs and acceptable LC rate, PBT is a feasible option for the treatment of lung oligometastasis in esophageal cancer patients.

## Introduction

The concept of an oligometastatic state, an intermediate state between limited primary and polymetastatic cancer, was first proposed by Weichselbaum and Hellman [1]. In cases involving an oligometastatic state, directed therapies, such as surgery and stereotactic body radiotherapy (SBRT), have the potential to prolong survival or cure the disease [2]. Even for lung metastases, prolonged life expectancy with metastasectomy has been demonstrated in various cancers [3,4].

In Japan, to receive public medical insurance coverage for SBRT for oligometastatic diseases, specific criteria introduced in April 2004 must be met: primary cancer is controlled and the patient has one to three lung oligometastases without external lung diseases. A systematic review reported that the clinical outcomes of lung metastases from various primary cancers with SBRT were comparable to those after surgery [5]. The one-, two-, and three-year overall survival (OS) rates after lung metastasectomy for esophageal cancer were 60%-100%, 36%-100%, and 40.2%-60%, respectively, in previous reports [6-11]. Moreover, the one-, three-, and five-year OS rates of SBRT for lung metastases of esophageal cancer in an additional report were 80.4%, 37.5%, and 23%, respectively [12]. A Japanese nationwide multicenter cohort study on particle therapy (PT) was initiated in May 2016 in all centers that performed proton beam therapy (PBT) and/or carbon ion beam therapy. In a 2023 cohort study using the same data to evaluate the effectiveness of these therapies for lung oligometastases, only eight patients had lung metastases from esophageal cancer out of 132 enrolled patients [13]. Furthermore, to date, there have been no reports on PBT for lung oligometastases in esophageal cancer.

In the present study, we retrospectively analyzed the feasibility and safety of PBT for recurrent lung oligometastases after esophageal cancer treatment.

## Materials and methods

Study design and patient enrollment

This single-center historical cohort study analyzed the feasibility and safety of PBT for oligometastatic lung recurrence in esophageal cancer. Patients who underwent PBT at our proton therapy center between 2010 and 2019 were selected from the database, based on the following criteria: the presence of primary esophageal cancer controlled by surgery or PBT with chemotherapy and the presence of one to three metachronous lung lesion recurrences without external lung diseases. Positron emission tomography with 2-deoxy-2-[fluorine-18] fluoro-D-glucose integrated with computed tomography (18F-FDG PET/CT) was performed on all patients to ensure that there were no tumors located outside the lungs. All metastatic tumors were lung oligometastatic recurrence after definitive treatment and primary cancer controlled.

Exclusion criteria

Stage IV cases with lung metastases from the beginning of the study period were excluded, as were cases in which the primary tumor’s pathology was unknown.

PBT procedure

Treatment indications were deliberated and determined by the cancer board. Radiation oncologists and medical physicists discussed the dose and fraction. Considering the location of the tumor and adjacent organ at risk, we modified the dose per fraction to 2 grays (relative biological effect)/fractions (Gy (RBE)/fr) and 74 Gy (RBE)/37 fr for the adjacent reconstructed gastric tube type, 2.4-3.3 Gy (RBE)/fr and 67.2-72.6 Gy (RBE)/22-28 fr for the adjacent hilum of the lung, and 6-8 Gy (RBE)/fr and 60-66 Gy (RBE)/8-10 fr for the peripheral lung area away from the hilum. We administered PBT one fraction per day, five days per week, Monday to Friday.

In the three-dimensional treatment planning procedure, chest computed tomography (CT) was performed at 2-mm intervals with respiratory gating with the patient in the supine position. Respiration gating was controlled by checking body movements using a laser rangefinder. The respiratory monitoring system AZ-733V (Anzai Medical, Tokyo, Japan) was used.

The gross tumor volume (GTV), defined as the volume of the lung tumor, was determined using CT and 18F-FDG PET/CT before treatment. The clinical target volume (CTV) was the GTV, with a margin of 5 mm in all directions. The planning target volume (PTV) was CTV plus 5-mm margins in all directions and 2-mm margins in the craniocaudal region depending on respiratory movements. The treatment planning system used was XiO-M (Hitachi, Kashiwa, Japan). For all patients who had previously undergone irradiation for primary esophageal tumors, safety in the present study was evaluated based on the cumulative dose of the previous irradiation and that for lung metastases. This was done by integrating the sites of the previous irradiation with those of the lung metastases. The calculations were performed using devices called RayStation (RaySearch Laboratories, Sweden) or Velocity (Varian Medical Systems, Palo Alto, CA). The proton beam irradiation method was passive scattering, which is a wobbler method. Hitachi’s proton-type particle therapy system (Hitachi, Kashiwa, Japan) was used for PBT. The beam energy and spread-out Bragg peak were fine-tuned to the target volume such that 90% of the isodose volume of the prescribed dose encompassed the planned target volume. The irradiation was administered while the patient was slowly breathing; however, the beam is on during the end-expiration phase using a breathing gate.

Outcomes

OS, progression-free survival (PFS), and local control (LC) rates and adverse events (AEs) were assessed. OS was the time from the date of PBT initiation to all-cause death at one-, two-, and three-year follow-ups. PFS was the time from the date of PBT initiation to disease progression and death. If no mortality event was observed, the case was terminated by censoring the date of the last follow-up. The date of the initial LC event was defined as the date when the response evaluation criteria in the solid tumor evaluation [14] resulted in progressive disease after the initiation of PBT.

Medical records were investigated to assess AEs according to the Common Terminology Criteria for Adverse Events version 5.0. During the PBT session, acute treatment-related toxicity was assessed and documented daily, and the patients were evaluated weekly using blood tests. After the completion of PBT, the patients were evaluated every three months by physical examination, blood tests, CT, and/or 18F-FDG PET/CT.

Statistical analysis

OS, PFS, and LC rates were calculated using the Kaplan-Meier method. Factors that may be related to OS, such as age, Charlson Comorbidity Index, tumor size (for multiple lesions, the size of the largest lesion), number of lung lesions, duration of primary tumor treatment to initiation of PBT, tumor-node-metastasis (TNM) classification, staging, post-PBT chemotherapy, and treatment of primary tumor, were investigated. The cutoff values ​​were estimated using the receiver operating characteristic curve and the area under the curve. The final cutoff value was selected as the point at which the sum of sensitivity and specificity was maximized. Univariate analysis was performed using log-rank tests. For significant factors, the mean of the two groups’ parameters for each factor was examined using a t-test to determine the absence of confounding factors within each group at the cutoff value. Statistical significance was set at p < 0.05. All statistical analyses were performed using EZR (Saitama Medical Center, Jichi Medical University, Saitama, Japan), a graphical user interface for R d4 (R Foundation for Statistical Computing, Vienna, Austria). EZR is a modified version of the R commander, designed to add statistical functions commonly used in biostatistics [15].

Ethical approval

All procedures involving human subjects performed in this study followed the ethical standards of the institutional and national research committees and the 1964 Declaration of Helsinki and its subsequent amendments. Informed consent or the substitute for it (as described below) was obtained from all patients included in the study. The study was announced on the website of our proton therapy center, and the contact information for those who declined to participate in the study were clearly indicated. We provided sufficient explanation that “no disadvantages will be incurred in the event of refusal to participate.” We explained that if no response was received within a certain period of notice, the participant would be deemed to have agreed to participate in the study. This study was approved by the ethics committee of Southern Tohoku General Hospital (approval number: 551).

## Results

This study included 11 males with a median age of 70 years (range: 59-71 years). Fifteen lesions from 11 patients were included. One patient received additional PBT for intrapulmonary recurrence after the previous PBT. The patient underwent additional PBT for one lesion. The most frequent dose was 60 Gy (RBE)/10 fr for four lesions, 66 Gy (RBE)/10 fr for four lesions, and 64 Gy (RBE)/8 fr for four lesions. The median time to PBT from primary esophageal cancer treatment, lesion size, fraction size, and biologically effective dose (BED) 10, were 23.7 months (7.2-71.9), 16 mm (6.1-28.3), 6.6 Gy (RBE)/fr (2-8), and 109.6 Gy (83.3-115.2), respectively. Table 1 summarizes patient and tumor characteristics. According to the eighth edition of the Union for International Cancer Control (UICC) TNM classification, five patients were T1, one was T2, two were T3, and three were T4; six were N0, three were N1, one was N2, and one was N3; and nine were M0 and two were M1. Regarding UICC staging, four patients were stage I, one was stage II, two were stage III, and four were stage IV. The pathological diagnoses were squamous cell carcinoma in 10 patients and basaloid (squamous) carcinoma in one patient. Seven patients had a lesion in the right lung, two in the left lung, and two in both lungs. Table 2 summarizes the details of irradiation and chemotherapy. Four (36.4%) patients received chemotherapy prior to PBT for lung metastasis; two (18.2%) chemotherapy concurrently with PBT, and six (54.5%) chemotherapy post-PBT.

**Table 1:** Patient and tumor characteristics Abbreviations: CCI, Charlson Comorbidity Index; T, tumor; N, lymph node; M, metastasis; UICC, Union for International Cancer Control; No., number; Ce, cervical esophagus; Ut, upper thoracic esophagus; Mt, middle thoracic esophagus; Lt, lower thoracic esophagus; L/R, left/right; R, right; L, left; S, segment; PBT, proton beam radiotherapy; SCC, squamous cell carcinoma; basaloid, basaloid (squamous) carcinoma, y/o: years old

Case	Age	CCI		Primary location	T	N	M	Stage UICC 8^th^ edition	Primary treatment	Pathology	No. of lesions	Location		Size (mm)
												L/R	Segment	
1	71 (y/o)		0	Lt	3	1	0	III	Surgery	SCC	3	R	S3	21
												L	S3	18.6
												L	S1+2	13.6
2	71 (y/o)		2	Mt	3	0	0	II	PBT	SCC	1	R	S2	6.1
3	59 (y/o)		0	Mt	2	2	0	III	Surgery	SCC	1	R	S9	17.6
4	62 (y/o)		0	Ce	4	1	1	IVB	PBT	SCC	1	R	S8	13.1
5	71 (y/o)		0	Mt	4	3	0	IVA	PBT	SCC	1	L	S3	16
6	70 (y/o)		0	Mt	1	0	0	I	Surgery	SCC	2	R	S3	28.3
												L	S9	15.2
											1	R	S1	10.3
7	60 (y/o)		0	Mt	1	0	0	I	Surgery	SCC	1	R	S2	16.4
8	60 (y/o)		0	Mt	1	0	1	IVB	PBT	SCC	1	R	S3	13.7
9	71 (y/o)		0	Mt	1	0	0	I	Surgery	Basaloid	1	R	S6	20.7
10	64 (y/o)		1	Mt	4	1	0	IVA	PBT	SCC	1	R	S9	11.4
11	71 (y/o)		0	Ut	1	0	0	I	Surgery	SCC	1	L	S6	23

**Table 2:** Characteristics of treatment and outcome Abbreviations: PBT, proton beam therapy; RBE, relative biological effectiveness; fr, fraction; PFS, progression-free survival; OS, overall survival; PTX, weekly paclitaxel; S-1, tegafur gimeracil oteracil potassium; DTX, docetaxel; CPT-11, irinotecan hydrochloride hydrate; CDDP, cisplatin; LN, lymph node; GY: gray

Case	Time to PBT from primary treatment (months)	Dose Gy (RBE)/fr	Chemotherapy			Local progression	Progression other site	PFS time (months)	OS time (months)	Cause of mortality	
			Precedent	Concurrent	Adjuvant						
											1	9.4	74/37	PTX	None	None	None	None	8.8	37.4	Alive	
		60/10				Exist					
		64/8				None					
2	12.7	64/8	None	CDDP	CDDP	None	None	19.7	19.7	Alive	
3	12.9	72.6/22	None	None	None	None	Lung/LN	2.1	11.9	Cancer	
4	7.2	60/10	None	None	None	None	None	8.8	8.8	pneumonia	
5	8.4	66/10	None	None	TS-1	Exist	Lung/LN	14.8	43.7	Cancer	
6	68.8	66/10	TS-1	None	None	None	Lung/LN	2.7	141.3	Alive	
		66/10				None					
	71.2	60/10				None					
7	22.4	60/10	DTX	None	TS-1	None	LN	4	15.9	Cancer	
8	25	66/10	None	TS-1	TS-1	Exist	Lung/LN	15.3	25.8	Cancer	
9	31.6	67.2/22	CPT-11+CDDP	None	TS-1	None	Lung/LN	3	27.8	Cancer	
10	43.2	64/8	None	None	None	None	None	49.2	49.2	Alive	
11	44.5	64/8	None	None	TS-1	None	None	38.2	38.2	Alive	

Survival outcomes

**Figure 1:**
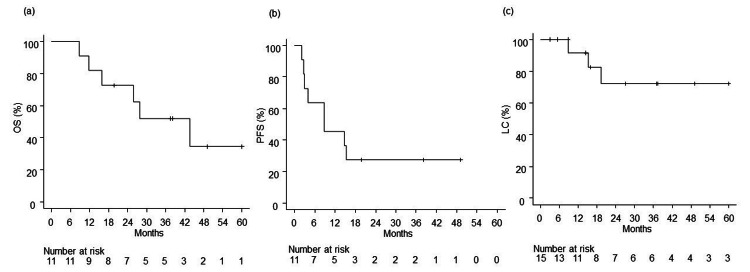
Kaplan-Meier plot of estimated OS, PFS, and LC: (a) OS and (b) PFS curves for all treated patients and (c) LC curves for all treated lesions Abbreviations: OS, overall survival; PFS, progression-free survival; LC, local control

The median follow-up period was 27.8 months (8.8-141.3 months). As of December 2022, six patients had died (including one death from infectious pneumonia). The one-, two-, and three-year OS rates were 81.8% (95% confidence interval (CI): 44.7%-95.1%), 72.7% (95% CI: 37.1%-90.3%), and 51.9% (95% CI: 19.8%-76.7%), respectively. The median survival time was 43.7 months. The one-, two-, and three-year PFS rates were 45.5% (95% CI: 16.7%-70.7%), 27.3% (95% CI: 6.5%-53.9%), and 27.3% (95% CI: 6.5%-53.9%), respectively. The median PFS time was 8.8 months. The one-, two-, and three-year LC rates were 92.3% (95% CI: 53.9%-98.8%), 72.7% (95% CI: 35.7%-90.2%), and 72.7% (95% CI: 35.7%-90.2%), respectively. The OS, PFS, and LC rates are shown in Figure 1 as Kaplan-Meier curves. Three patients had lesions that were local recurrences; one patient had the lesion surgically removed, one was treated with immunotherapy, and one was observed. New lung and lymph node metastases were observed in five patients, and one was treated with PBT. Additional PBT was performed safely without complications.

OS-related factors

Age (p = 0.01) and metastases in the TNM classification of the primary cancer, which includes distant metastatic lymph node metastases only (p = 0.03), were significant OS-related factors. A summary of the univariate analysis is presented in Table 3. Regarding age, using a cutoff of 62 years, the size of the tumors, number of lesions, and duration of primary tumor treatment to the initiation of PBT were compared for the mean of each group; t-tests were used to analyze the means. None of the variables showed significant differences between the two groups (<62 and ≥62). The <62 group had two patients with distant lymph node metastasis, and the ≥62 group had no patients with distant lymph node metastasis. The results of the t-tests are presented in Table 4.

**Table 3:** Summary of univariate analysis results Abbreviations: AUC, area under the curve; CI, confidence Interval; n, number of patients; MST, median survival time; CCI, Charlson Comorbidity Index; PBT, proton beam therapy; PFS, progression-free survival; NA, not available; T, tumor; N, lymph node; M, metastasis

Factor	Median	Range	Cutoff value	AUC	95% CI	Group	n	MST	p value
Age	70	59-71	62	0.767	0.467-1	<62	3	15.9	0.01
						≥62	8	43.7	
CCI	0	0-1	0	0.3	0.06-0.54	<1	9	27.8	0.20
						≥1	2	NA	
Size of tumors	16 mm	6.1-28.3	13.1	0.4	0-0.88	<13.1	3	NA	0.74
						≥13.1	8	35.75	
Number of lesions	1	1-3	1	0.3	0.06-0.54	<2	9	27.8	0.14
						≥2	2	NA	
Duration of primary tumor	23.7 months	7.2-71.9	12.9	0.267	0-0.63	<12.9	5	43.7	0.46
Treatment to initiation of PBT						≥12.9	6	27.8	
PFS time after PBT	8.8 months	2.1-49.2	3	0.25	0-0.60	<3	3	27.3	0.82
						≥3	8	43.7	
T	2	1-4	4	0.467	0.11-0.83	<4	8	27.8	0.99
						≥4	3	43.7	
N	0	0-3	2	0.617	0.29-0.94	<2	9	NA	0.36
						≥2	2	27.8	
M	0	0-1	1	0.667	0.46-0.87	<1	9	43.7	0.03
						≥1	2	17.3	
Stage	3	1-4	4	0.633	0.29-0.96	<4	7	NA	0.62
						≥4	4	34.75	
Prior	-	-	-	-	-	Presence	4	27.8	0.70
Chemotherapy	-	-	-	-	-	Absence	7	43.7	
Concurrent	-	-	-	-	-	Presence	2	25.8	0.75
Chemotherapy	-	-	-	-	-	Absence	9	43.7	
Post-PBT	-	-	-	-	-	Presence	6	27.8	0.51
Chemotherapy	-	-	-	-	-	Absence	5	NA	
Primary tumor treatment	-	-	-	-	-	Surgery	6	27.8	0.82
	-	-	-	-	-	PBT	5	43.7	

**Table 4:** Summary of t-test results Abbreviations: SD, standard deviation; PBT, proton beam therapy; PFS, progression-free survival; LN, lymph node, No., number; y/o, years old

Variables	Age	Mean	SD	p value
Size of tumors	<62 (y/o)	15.9 (mm)	2.00	0.73
	≥62 (y/o)	17.5 (mm)	7.15	
Number of lesions	<62 (y/o)	1	0.00	0.42
	≥62 (y/o)	2	0.74	
Duration of primary tumor	<62 (y/o)	20.1 (months)	6.37	0.57
Treatment to initiation of PBT	≥62 (y/o)	28.2 (months)	22.6	
PFS time after PBT	<62 (y/o)	7.14 (months)	7.14	0.317
	≥62 (y/o)	17 (months)	17.0	
		No. of patients		
Distant metastatic LN	<62 (y/o)	2		
	≥62 (y/o)	0		

Safety outcomes

No grade ≥ 3 radiation-induced AEs were observed. None of the patients experienced acute radiation pneumonitis. Acute AEs only included grade 1 radiation-induced dermatitis (n = 4; 36.4%). The observed late AEs were grade 1 radiation-induced rib fracture (n = 2; 18.2%) and grade 1 radiation pneumonitis (n = 9; 81.8%).

## Discussion

The present study showed satisfactory OS and LC rates for lung oligometastases in esophageal cancer patients. The OS rates are comparable to those of lung resection (one-year OS rates: 60%-100%, two-year OS rates: 36%-100%, three-year OS rates: 40.2%-60%) in previous reports [6-11]. A Japanese nationwide multi-institutional retrospective analysis of PBT for one to three lung oligometastases from various cancers reported one-, two-, and three-year LC rates of 92.2%, 86.3%, and 78.4%, respectively [16]. Local treatment results for lung metastases of esophageal cancer are summarized in Table 5. Although the number of cases in the present study was small, the one-, two-, and three-year LC rates of the current study were 92.3%, 72.7%, and 72.7%, respectively. The results of the present study are comparable with those of previous studies. To the best of our knowledge, there are no reports of PBT for lung metastases in esophageal cancer; therefore, we consider our findings valuable and worthwhile.

**Table 5:** Summary of local treatment results of lung metastasis from esophagus cancer Abbreviations: PBT, proton beam therapy; MST, median survival time; AEs, adverse events; NA, not available; *, including gastric cancer; SBRT, stereotactic body radiotherapy

Authors	Country	Year	Treatment	Number of patients	Type of lesion	Survival rate (%)			MST (months)	AEs
						1-year	2-year	3-year		
Takemura et al. [6]	Japan	2012	Surgery	5	≤1	60	36	NA	NA	NA
Ichida et al. [19]	Japan	2013	Surgery	5	≤2	80	80	60	48	NA
Kobayashi et al. [20]	Japan	2014	Surgery	23	≤3	82.6	NA	46	37.4	NA
Kozu et al. [7]	Japan	2015	Surgery	15	≤2	93	NA	44	32	G3 ≤ 33%
Kanamori et al. [8]	Japan	2017	Surgery	33	≤3	79.4	NA	47.8	17.9	G3 ≤ 15%
Komatsu et al. [9]	Japan	2019	Surgery	16	Multiple	93.8	NA	40.2	NA	G3 ≤ 9%
Seesing et al. [10]	Netherlands	2019	Surgery	4^* ^+ 11	≤2	67	53	53	Not reached	G3 ≤ 27%
Lo et al. [11]	Taiwan	2022	Surgery	14	≤5	100	100	NA	Not reached	0%
Yamamoto et al. [12]	Japan	2020	SBRT	114	≤5	80.4	NA	37.5	27.1	G3 ≤ 4.4%
Current study	Japan	2023	PBT	11	≤3	81.8	72.7	51.9	43.7	G2 ≤ 0%

Furthermore, we demonstrated the absence of grade 3 or higher AEs. A previous study on PBT for lung oligometastases in various primary cancers reported seven patients with grade 2 AEs (5.9%) and one with grade 3 AEs of radiation dermatitis (0.8%) [16]. In a study on lung oligometastases in esophageal cancer, three (3.3%) patients had grade 2 AEs, four (4.4%) had grade 3 or higher AEs, and one (1.1%) had grade 5 radiation-induced pneumonitis [12]. A Japanese nationwide survey of SBRT for lung oligometastasis reported AEs in 1,040 patients and grade 2, 3, 4, and 5 pneumonitis in 96 (9.2%), 14 (1.3%), 2 (0.1%), and 7 (0.6%) patients, respectively. Grade 5 hemoptysis occurred in three (0.2%) patients [17]. In contrast, grade 3 or higher AEs in lung resection have been reported in 9%-33% of all reported cases [7-10]. The current study included two patients with tumors in the hilum of the lung. If a tumor in the hilum was resected, it would be by enlarged surgery. This type of tumor can be treated safely in a minimally invasive manner using a PBT dose protocol of 72.6 GyE (RBE)/22 fr. Two patients had tumors in both lungs that were treated simultaneously without any AEs.

Kroese et al. reported a systematic review and meta-analysis of oligometastatic esophagogastric cancer in which local oligometastasis-directed treatments, such as metastasectomy, SBRT, and brachytherapy, improved OS compared with systemic therapy alone. Esophagogastric cancer spread limited to one organ with ≤3 metastases or one extra-regional lymph node situation was considered oligometastatic disease [18]. Lung oligometastases are rare in patients with esophageal cancer. Ichida et al. reported that only five (1.7%) patients with postoperative primary esophageal cancer underwent pneumonectomy [19]. In addition, Kobayashi et al. reported that of 679 patients who underwent postoperative or definitive chemoradiation therapy for primary esophageal cancer, 23 (3.4%) underwent pneumonectomy, and very few underwent pneumonectomy [20].

In the Stereotactic Ablative Radiotherapy for the Comprehensive Treatment of Oligometastases (SABR-COMET) phase II randomized trial, patients with various controlled primary cancers and one to five metastatic lesions were treated for metastatic lesions with SBRT, which improved OS compared to palliative chemotherapy (p = 0.006) [21]. The SBRT-COMET study did not include lung metastases from esophageal cancer. However, the SBRT-COMET study reported that the addition of local therapy using radiotherapy for a small number of metastases (≤5) in addition to standard chemotherapy can prolong life. In Japan, the SABR-COMET study is an important paper reporting evidence for public insurance coverage of SBRT for oligometastases. It is impossible to resect many lesions; therefore, surgical reports may be limited to a small number of lesions. Thus, the use of minimally invasive therapies such as radiotherapy, including SBRT or PT, might be an extended indication of oligometastasis-directed treatment for esophageal cancer patients.

The PEMBRO-RT phase II randomized clinical trial reported that the SBRT on a single tumor site preceding pembrolizumab treatment enhanced tumor response in patients with metastatic non-small cell lung cancer (NSCLC). The median survival time of the immune checkpoint inhibitor (ICI) plus irradiation group was prolonged twofold compared to that in the ICI alone group [22]. In Japan, the ICI is covered by public insurance for the recurrence of esophageal cancer patients. The ICI and PBT combination therapy is expected to have further synergistic effects on the suppression of recurrent lung metastases from esophageal cancer.

We previously reported PBT for liver oligometastatic recurrence in patients with esophageal, gastric, or colorectal cancer. The one-, two-, and three-year LC rates in all cases were 76.9%-100%, 54.9%-100%, and 54.9%-100%, respectively. The median liver metastatic lesions from the esophageal, gastric, and colorectal cancers were 22.6 mm (7-55 mm), 31 mm (13-68 mm), and 30 mm (9-103 mm), respectively [23-25]. SBRT, which is another local radiotherapy option in terms of safety and repeatability, is limited to tumors of ≤5 cm in diameter. In Japan, SBRT for liver metastasis is covered by public health insurance, although it is also limited to tumors of ≤5 cm. An advantage of PBT is to treat much larger tumors without severe adverse effects. Unfortunately, the present study had a median tumor size of 16 mm (6.1-28.3 mm), and they can be treated with SBRT. This is because, unlike liver metastases, lung metastases are easily detected on follow-up CT when they are relatively small and/or are at an early stage, and this is the difference between lung metastasis and liver metastasis, which is difficult to detect using CT.

There are two reports of PBT for liver metastases of esophageal cancer. The total number of cases in these reports was very small (13 cases), but the one-, two-, and three-year LC rates were 100% for all [23,26]. We had thought that both lung and liver metastatic lesions from esophageal cancer would be considered equally sensitive to PBT. However, the one-, two-, and three-year LC rates in the present study were 92.3%, 72.7%, and 72.7%, respectively, and three cases showed local recurrence. The causes were investigated, but not well understood. Management of respiratory movement of targets in case of lung metastasis from esophageal cancer may need to be inspected.

The present study has several limitations. First, because the study was performed at a single institution, the sample size was relatively small. Similarly, the 2023 Japanese cohort study on lung oligometastases described earlier in the Introduction section included only eight patients with lung metastasis from esophageal cancer out of 132 patients enrolled for lung oligometastasis treatment [13]. The limitations of the small number of patients could not be resolved. It may need to be widely recognized that lung oligometastasis is an indication for local treatments such as PBT. Second, the current study had no control group; therefore, we were unable to perform comparisons with other standard treatments. However, comparing surgery with radiotherapy is not realistic, because the approaches are very different. Therefore, more reports on the outcomes of radiotherapy are necessary to enable meta-analyses. Third, we treated patients with PBT who were referred from other hospitals. Thus, we do not know how many patients with esophageal cancer were eligible for PBT for lung metastasis recurrence. Therefore, our results cannot be generalized.

## Conclusions

Based on the low incidence of AEs and acceptable LC rate, we found that PBT is a feasible option for the treatment of lung oligometastasis in esophageal cancer patients. However, the standard treatment for esophagus cancer with metastases is chemotherapy. Therefore, it is not clear whether PBT is a better option for esophageal cancer patients with oligometastasis, because the results of the present study are not clear on whether the prognosis improved. Further study is necessary to clarify whether local treatment, such as PBT, in addition to systemic chemotherapy improves the prognosis of esophageal cancer patients with lung oligometastatic recurrence.
